# A prospective study for an alternative probe site for pulse oximetry measurement in male patients with severe burn trauma: penile shaft

**DOI:** 10.55730/1300-0144.5610

**Published:** 2023-02-02

**Authors:** Yeşim ŞENAYLI, Gülsen KESKİN, Mine AKIN, Atilla ŞENAYLI, Rabia ATA, Gökhan DEMİRTAŞ, Emrah ŞENEL

**Affiliations:** 1 Department of Anesthesia and Reanimation, School of Medicine, Bozok University Yozgat Turkey; 2 Department of Anesthesia and Reanimation, Ministry of Health Ankara City Hospital Ankara, Turkey; 3 Department of Pediatric Surgery, School of Medicine, Bozok University, Yozgat, Turkey; 4 Department of Pediatric Surgery, Çam ve Sakura City Hospital İstanbul, Turkey; 5 Department of Pediatric Urology, Ministry of Health Ankara City Hospital, Ankara, Turkey; 6 Department of Pediatric Surgery, School of Medicine, Ankara Yıldırım Beyazıt University Ankara, Turkey

**Keywords:** Penile, probe, children, burns, pulse oximetry

## Abstract

**Background/aim:**

Authors widely use pulse oximetry in clinical monitoring of heart rate (HR) and peripheral oxygen saturation (SpO_2_) by attachment to the fingers; however, there can be a need for an alternative attachment site, especially for burned patients. We investigate the availability of a pulse oximeter probe attached to the penile shaft as an alternative site in pediatric male patients if all extremities became unavailable for pulse oximetry measurement due to severe burn and/or trauma.

**Materials and methods:**

We designed a prospective comparative study in a training and research hospital. After local ethical committee approval, pediatric male cases eligible for penile and extremity pulse measurements were evaluated during general anesthesia for medical dressing and/or grafting due to severe burns. One probe was attached to the fingers of the unburned extremity, and the other was to the penile shaft. Furthermore, we recorded SpO_2_ and HR values at 5-min intervals; 0th (baseline), 5th, 10th and 15th minutes. We compared HR and SpO_2_ values measured by the finger probe with those measured by the penile probe.

**Results:**

Data of 51 patients (median age, 2.9 years (interquartile range, 2.0–5.0 years)) in whom the duration of dressing was at least 15 min were analyzed. There was no significant difference either in comparisons of hemodynamic measurements (HR and SpO_2_) obtained by finger probe and by a penile probe for each measurement time. The Bland-Altman plot analysis reveals agreement for penile and finger probes with a mean bias value between 0.20 and 0.37 on HR and between 0.43 and −0.20 on SpO_2_.

**Conclusion:**

This clinical trial demonstrated that pulse oximetry measurement under nonhypoxic conditions we could perform confidently using penile probes in pediatric male patients whose extremities are unavailable for measurement.

## 1. Introduction

Arterial blood oxygen saturation (SaO2) has clinical and physiological importance. It is a parameter we must closely monitor in many patients as a direct indicator of oxygen supply to the organs and tissues. SaO2 level can be determined noninvasively by pulse oximetry; this measurement, defined as peripheral oxygen saturation SpO_2_, has been reported to provide an accurate result with a standard deviation (SD) of 2% compared with the invasive method [1]. As a rational and easily applicable method, pulse oximetry is widely used in clinical practice for monitoring heart rate (HR) and SpO_2_. Pulse oximetry measurement may reduce the frequency of hypoxemia by guiding oxygen therapy.

Consequently, intensive care unit admissions might decrease. It may reduce patients’ arterial blood sampling by allowing noninvasive monitoring [2]. Studies on developing new-generation devices and implementations have been conducted in this field [3–6].

Pulse oximeter sensors are usually attached to the fingers; however, there is a need for an alternative site for the sensors to be connected. If peripheral perfusion is inadequate or the usual extremities for pulse oximetry measurement are unavailable, especially in trauma involving extremity injuries, finding a good body side to place pulse oximetry might be difficult. There could be a problem with a body area where a safe pulse oximetry probe can be placed.

The present study aimed to investigate the availability of a pulse oximeter probe attached to the penile shaft as an alternative site in male patients. In the literature, a study on burned children evaluating an alternative pulse oximetry side like a penis is not present. However, in practice, anesthesia staff can deal with a patient with four extremity injuries. This study will help anesthetists remodel their practice for these patients. Assuming that extremities became unavailable for pulse oximetry measurement, burned patients were evaluated for their availability at a different measurement site, especially by a penile shaft.

## 2. Materials and methods

Clinical Research Ethics Committee of Ankara Child Health and Disease, Hematology, Oncology Training, and Research Hospital, Ankara, Turkey (Chairperson Prof. Fatma Demirel) on 01 June 2015 (Approval number: 2015–025) approved the present study. The study was conducted in Ankara Child Health and Disease, Hematology, Oncology Training, and Research Hospital, Ankara, Turkey. Informed consent of the patients or the legal guardians was also obtained. The study was conducted between September 2015 and March 2017.

Pediatric male cases undergoing general anesthesia for medical dressing and/or grafting due to severe burns were involved in the study. We included patients on the condition that the fingers of at least one extremity were not involved in burn injury or a surgical procedure. Exclusion criteria were only performed for burned children with cardiovascular diseases as pulse oximetry measurements were compared within the patients’ extremities and penis. Possible differences in pulse measurements between these locations are taken into account. However, we had no cardiovascular patients with burn injuries.

Monitoring via pulse oximetry measurement was initiated as soon as the patients were placed on the operating table. Monitoring was performed using two pulse oximetry devices with the same brand and model (Nellcor N-560 Pulse Oximeter Monitor, Mansfield, USA). Moreover, two disposable oximeter probes (OxiMax Technology Max-N-Infant/Adult SpO_2_ Sensor, Mansfield, USA) were used. We attached one of the probes to the finger of the unburned extremity, while the other was attached to the penile shaft. We noted the finger the probe was attached to and ensured that the sphygmomanometer cuff was not wrapped around the same extremity. We recorded each probe’s results and measured SpO_2_ and HR values at 5-min intervals for each probe starting from baseline time (0th) and continuing with the 5th, 10th and 15th minutes. HR and SpO_2_ values measured by the finger probe were compared with those measured by the penile probe.

### 2.1. Statistical analysis

Data analysis was performed using MedCalc version 11.1.1.0 (MedCalc Software, Mariakerke, Belgium). Where appropriate, data were expressed as mean± SD or median (interquartile range [IQR]). The mean differences in hemodynamic measurements (i.e. HR and SpO_2_) between finger and penile probes were compared using paired samples t-test. The repeated measures of analysis of variance (ANOVA) by Wilks’ lambda test were applied to determine whether the differences in hemodynamic measurements among measurement times were statistically significant or not. Wilcoxon signed-rank test was used to evaluate any significant difference between percentage changes of hemodynamic measurements by finger probe and penile probe at measurement times concerning baseline. Bland-Altman plots were used to assess agreement between penile and finger probes in HR and SpO_2_ measurements. The means of agreement differences (i.e. bias), SDs of bias levels, and upper and lower limits at 95% confidence interval were also calculated. p value less than 0.05 was considered statistically significant.

## 3. Results

The study included 56 male pediatric patients with severe burns. There were no race or ethnicity differences. As the medical dressing lasted for 5 min in 2 cases and 10 min in 3 cases, these cases were excluded, and thereby data of 51 patients in whom the duration of dressing was at least 15 min were analyzed. These 51 patients had a median age of 2.9 years (IQR, 2.0–5.0 years) and a median body weight of 15 kg (IQR, 12.0–21.0 kg). Of these 51 patients, 38 had a dressing duration of 20 min. At the 0th minute lowest value of SpO_2_ levels were 95, and highest value was 100. Also at the 0th minute baseline value was 98. At the 5th minute lowest value of SpO_2_ levels were 96, and highest value was 100. Also at the 5th minute baseline value was 98. At the 10th, follow-up values were seemed to be similar with 5th minute lowest value of SpO_2_ levels were 96, and highest value was 100. Also at the 10th minute baseline value was 98. At the 15th minute lowest value of SpO_2_ levels were 98, and highest value was 100. Also at the 15th minute baseline value was 99.

In 51 patients, there was no significant difference either in comparisons of hemodynamic measurements (HR and SpO_2_) obtained by finger probe and by a penile probe for each simultaneous measurement time (baseline and at the 5th, 10th, and 15th minutes of dressing) or in comparisons of hemodynamic measurements among measurement times for finger or penile probes shown in Table 1. We used left hand fingers in 11 patients. Also, we used right hand, right foot and left foot fingers in 12, 14 and 14 patients, respectively.

No significant differences were determined between the percentage changes of HR measurements obtained by finger probe and penile probe at the baseline›s 5th, 10th, and 15th minutes. Likewise, there were no significant differences between the percentage changes of SpO_2_ measurements obtained by the finger probe and the penile probe at the 5th, 10th, and 15th minutes to baseline shown in Table 2.

**Figure 1:**
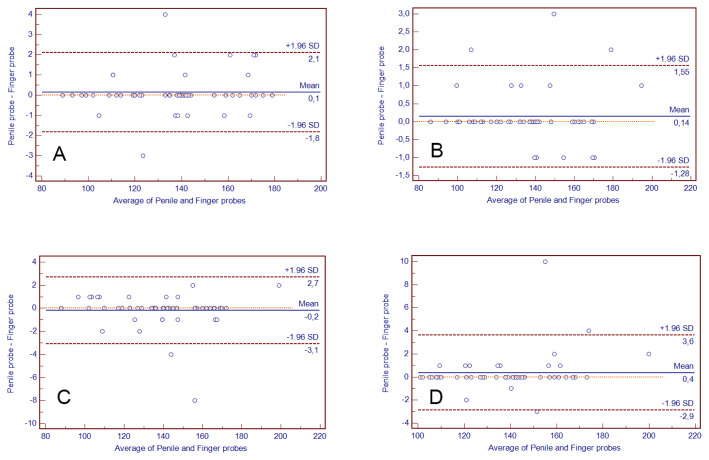
Bland-Altman plot showing the magnitude of the difference between heart rate (HR) obtained by penile and finger probes (n = 51). Heart rates unit is beat /minute. Solid lines indicate mean bias and dashed lines indicate the lower and upper limits of agreement. A: 0th (baseline) minute, B: 5th minute, C: 10th minute, and D: 15th minute.

**Figure 2:**
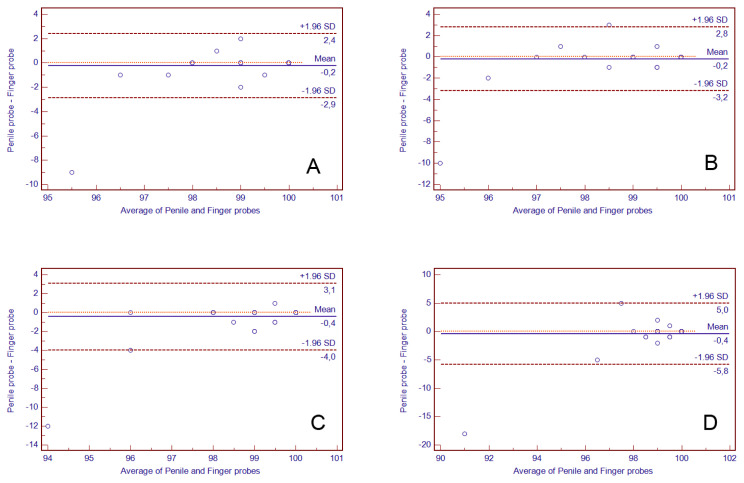
Bland-Altman plot showing the magnitude of the difference between oxygen saturation (SpO_2_) obtained by penile and finger probes (n = 51). The SpO_2_ unit is percent (%). Solid lines indicate mean bias and dashed lines indicate the lower and upper limits of agreement. A: 0th (baseline) minute, B: 5th minute, C: 10th minute, and D: 15th minute.

The results of the Bland-Altman plot analysis performed to assess the agreement between the penile and finger probes for the measurements of HR and SpO_2_ are presented in Figures 1 and 2, respectively. The mean bias value was between 0.20 and 0.37 for HR and between 0.43 and −0.20 for SpO_2_. The numerical outcomes of the analysis are demonstrated in detail in Table 3.

## 4. Discussion

In this study, we aimed to evaluate an alternative side of monitoring to follow up oxygen saturation in certain conditions. Having pulse oximetry results on the extremities might be a problem for burned children. Although the possibility of this situation is slight, it is not negligible. There for, we evaluate the penile shaft as a possible alternative side of monitoring. As hypothesized, the penis could be an alternative side of pulse monitoring if circumstances for limbs are unavailable.

In many clinical practices, pulse oximetry measurement is used for monitoring HR and SpO_2_. For the comfortable use of the pulse oximeter, it must show whether the patient has hypoxia by giving fast and highly appropriate results and that this feature is sustainable [7,8]. These practices may include general anesthesia [9], neonatal resuscitation [10,11], dental pulp vitality testing [12], and exercise in patients with the chronic obstructive pulmonary disease [13]. Pulse oximeter monitoring is cheap and easy to apply without time consumption with relatively high sensitivity. Therefore, pulse oximeter monitoring is indispensable in evaluating and managing and can be used even in clinical predictions of the trauma patient [14]. In general, pulse oximetry is performed using a finger probe; however, there may be a need for an alternative site for the sensors to be attached to in some circumstances when the extremities are unavailable for various reasons. In the literature, there are studies performing pulse oximeter measurements from different sites like nose [15–17], ear lobe [15,18], ear canal [19], buccal region [20], forehead [13,15, 21], and esophagus [22].

In studies with earlobes, it has been determined that pulse oximetry values are more accurate than finger and toe measurements [7]. In addition, it was determined that the measurements of the earlobe and the finger of the hand were compared and in patients who underwent fiberoptic bronchoscopy under anesthesia, oximeters working with the reflection mechanism were more effective than those working with the transmission method [23]. In another study conducted with the finger, it was stated that the fingers should be unfolded for proper results [7]. In a study comparing forehead and finger measurements, it was shown that there was no difference even in general anesthesia [7]. It has been shown that the measurements in the earlobe, finger, toe and forehead regions of patients who underwent coronary artery bypass grafting are compatible and can be used if necessary [7]. There are publications suggesting that palms, soles, penis, nasal wings, knee and elbow parts can also be used in infants [8]. In a study in which the knee and elbow parts were evaluated, it was stated that the localization where the best measurement would be made was the part with the best pulsatile vascular bed [8]. In the same study, it was stated that palm, sole, knee and elbow applications were not different from each other [8].

Robertson and Kaplan reported the penile shaft as a good site for a pulse oximeter probe [24]. They compared the measurements obtained from a probe attached to the penile shaft with those obtained by a probe attached to the nose in a pediatric male patient and reported SpO_2_ readings obtained from the penile shaft to be within 1%–2% of those obtained concurrently with the probe placed on the patient’s nose [24]. They also reported that the accuracy of the penile pulse oximetry values was confirmed by the analyses of several arterial blood samples [24].

In the present study, we investigated the availability of a pulse oximeter probe attached to the penile shaft in the event extremities were unavailable. Evaluation of the agreement between the measurements performed by each probe using Bland-Altman plot analysis revealed the mean bias value in 51 patients. Accordingly, based on the acceptable degree of bias and limits of agreement, we could conclude that the oximeter probe can be attached to the penile shaft when necessary. In a multicenter, observational study conducted on children, arterial blood SaO2 values were compared with pulse oximetry SpO_2_ values [25]. A total of 1980 simultaneous measurements were evaluated, and the bias (SpO_2_-SaO2) was reported to vary through the range of SpO_2_ values. The most significant bias was reported in the SpO_2_ range of 81% to 85%. Furthermore, SpO_2_ measurements were reported to be close to SaO2 in the SpO_2_ range from 91% to 97% [24]. Considering that the reasons that may cause bias, such as race, and chronic diseases, will cause significant differences in pulse oximetry measurements, no differences were observed in these levels in our study. In this case, bias should not be expected in patients treated with penile pulse oximetry measurement. Based on these results, the precision (SD) and accuracy were considered poor when SpO_2_ was <90% [24]. To express it as high accuracy, the difference between arterial blood gas analysis and SaO2 level should be at most 2%, and SaO2 levels should be between 70% and 100% [7]. However, physiological, environmental, technological, and human-induced problems may affect this accuracy [8]. In addition, a comparison was made with a single extremity in terms of precise pulse measurement because there was no damage to the limbs and penile probe application areas in the compared limbs of the patients, despite their burns. The fact that the measurements were at sure normality and stability eliminated the need for an additional third or fourth simultaneous measurement.

In the present study, the absence of comparison with arterial blood sample levels could be regarded as a limitation; nevertheless, in light of the data mentioned above, the comparisons could be considered to have acceptable accuracy since all SpO_2_ measurements were >90% in the present study. There are some other limitations of our study. Firstly, the study was designed assuming all four extremities are not available for probe measurements, but this condition is rare among burned patients. Therefore, it might not be a prior choice to evaluate a patient’s pulse oximetry in practical usage. Anaesthesia staff might prefer auricle or some other locations for this purpose. As it might differ according to clinics, another study evaluating this type of preference could be designed. In our study, as we decided to evaluate the penile effectiveness of probe usages, we did not study priority rankings of pulse locations. Our second limitation was demonstrating possible skin pathologies developed by the probe on the penile shaft. Although we did not observe skin problems during our study, much more patients had to be analyzed to say probes are harmless. Therefore, no significant adverse event associated with the penile probes was observed during the operation; the penile probe was observed directly by the anesthesia team and the surgical team during the process.

In conclusion, we suggest that pulse oximetry measurements during a medical emergency, intensive care services management, and peroperative period could be performed using penile probes in pediatric male patients whose extremities are unavailable for measurement in cases like trauma.

## Figures and Tables

**Table 1:** Pulse oximetry measurements using finger and penile probes in the course of dressing.

	Finger probe n = 51	Penile probe n = 51	p*
**Heart rate, beats/minute**			
Baseline	136.9 ± 23.5	137.0 ± 23.7	0.332
5th minute	136.7 ± 24.2	136.9 ± 24.2	0.180
10th minute	139.1 ± 23.7	138.9 ± 23.5	0.345
15th minute	137.9 ± 21.6	138.3 ± 22.	0 0.115
**p** **	0.411	0.616	
**Oxygen saturation, %**			
Baseline	99.6 ± 0.8	99.4 ± 1.5	0.258
5th minute	99.6 ± 0.9	99.4 ± 1.6	0.362
10th minute	99.6 ± 0.8	99.1 ± 2.0	0.094
15th minute	99.5 ± 0.9	99.1 ± 2.6	0.268
**p** **	0.948	0.196	

Data are presented as mean ± standard deviation.

* Paired samples t-test; comparisons of hemodynamic measurements obtained by finger probe and by penile probe for each measurement time.

** Repeated measurements of ANOVA by Wilks’ lambda test; comparisons of hemodynamic measurements among measurement times for finger or penile probe.

**Table 2:** Percentage differences in pulse oximetry measurements at the 5th, 10th, and 15th minutes with respect to baseline.

	Finger probe n = 51	Penile probe n = 51	p*
	Percentage change	Percentage change	
**Heart rate, beats/min**			
5th minute - Baseline	−0.7 (−2.8 – 1.7)	−0.7 (−2.8 – 2.2)	0.715
10th minute - Baseline	0.0 (−2.8 – 7.1)	0.0 (−3.5 – 6.5)	0.482
15th minute - Baseline	0.0 (−4.2 – 7.9)	0.0 (−4.2 – 7.4)	0.503
**Oxygen saturation, %**			
5th minute - Baseline	0.0 (0.0 – 0.0)	0.0 (0.0 – 0.0)	0.916
10th minute - Baseline	0.0 (0.0 – 0.0)	0.0 (−1.0 – 0.0)	0.172
15th minute - Baseline	0.0 (0.0 – 0.0)	0.0 (−1.0 – 0.0)	0.505

Data are presented as interquartile range.

* Wilcoxon signed-rank test; comparisons of percentage changes of hemodynamic measurements obtained by finger probe and by penile probe at the 5th, 10th, and 15th minutes with respect to baseline.

**Table 3:** Results of Bland-Altman analysis (n = 51).

	Mean bias	SD	Limits of agreement	
			LL	UL
**Heart rate**				
Baseline	0.14	1.00	−1.82	2.10
5th minute	0.14	0.72	−1.28	1.55
10th minute	−0.20	1.47	−3.08	2.68
15th minute	0.37	1.66	−2.88	3.63
**Oxygen saturation**				
Baseline	−0.22	1.35	−2.85	2.42
5th minute	−0.20	1.52	−3.18	2.79
10th minute	−0.43	1.80	−3.97	3.10
15th minute	−0.43	2.75	−5.82	4.96

Bias, differences between penile and finger probe measurements; SD, standard deviation; LL, lower limit; UL, upper limit.
